# Sortilin is dispensable for secondary injury processes following traumatic brain injury in mice

**DOI:** 10.1016/j.heliyon.2024.e35198

**Published:** 2024-07-29

**Authors:** Irina Staib-Lasarzik, Christina Gölz, Wieslawa Bobkiewiecz, Pawit Somnuke, Anne Sebastiani, Serge C. Thal, Michael K.E. Schäfer

**Affiliations:** a Department of Anesthesiology, University Medical Center of the Johannes Gutenberg-University Mainz, Mainz, Germany; b Department of Anesthesiology, Faculty of Medicine Siriraj Hospital, Mahidol University, Bangkok, 10700, Thailand; c Focus Program Translational Neurosciences (FTN) of the Johannes Gutenberg-University Mainz, Mainz, Germany; d Research Center for Immunotherapy, University Medical Center, Johannes Gutenberg-University Mainz, Mainz, Germany

## Abstract

Traumatic brain injury (TBI) is characterized by complex secondary injury processes involving the p75 neurotrophin receptor (p75NTR), which has been proposed as a possible therapeutic target. However, the pathogenic role of the p75NTR co-receptor sortilin in TBI has not been investigated. In this study, we examined whether sortilin contributes to acute and early processes of secondary injury using a murine controlled cortical impact (CCI) model of TBI. Initial expression analysis showed a down-regulation of sortilin mRNA levels 1 and 5 day post injury (dpi) and a reduced expression of sortilin protein 1 dpi. Next, a total of 40 Sortilin^ΔExon14^ loss-of-function mouse mutants (Sort1^−/−^) and wild-type (Sort1^+/+^) littermate mice were subjected to CCI and examined at 1 and 5 dpi. Neither sensorimotor deficits or brain lesion size nor CCI-induced cell death or calcium-dependent excitotoxicity as evaluated by TUNEL staining or Western blot analysis of alpha II spectrin breakdown products were different between Sort1^−/−^ and Sort1^+/+^ mice. In addition, CCI induced the up-regulation of pro-inflammatory marker mRNA expression (*Il6*, *Tnfa*, *Aif1*, and *Gfap*) irrespectively of the genotype. Similarly, the mRNA expressions of neurotrophins (*Bdnf*, *Ngf*, *Nt3)*, VPS10P domain receptors others than sortilin (*Ngfr, Sorl1*, *Sorcs2*), and the sortilin interactor progranulin were not affected by genotype. Our results suggest that sortilin is a modulatory rather than a critical factor in the acute and early brain tissue response after TBI.

## 1 Introduction

Traumatic brain injury (TBI) is a leading cause of morbidity and mortality worldwide [1]. There is a lack of effective treatment for TBI, probably owing to the complexity of the pathophysiological processes. These processes include, among others, defective cerebral edema and cerebral autoregulation, blood-brain barrier disruption, oxidative stress, mitochondrial dysfunction and different modes of neuronal cell death of neurons, that are always accompanied by robust responses of the innate immune system [[2], [3], [4], [5], [6]]. Consequently, therapeutic options are limited to surgical interventions and supportive therapies [[7], [8], [9]]. Therefore, further research is required to better understand the pathogenic mechanisms and to pinpoint molecular targets for novel therapeutic approaches.

Previous approaches demonstrated that the downstream signaling of neurotrophins plays an important role in TBI pathogenesis. While mature neurotrophins support neuronal survival in the adult brain [10], the precursors of neurotrophins, such as pro-nerve growth factor or pro-brain-derived neurotrophic factor were shown to promote neuronal cell death during development and following injury in the central nervous system [11]. Neurotrophins signal through at least two types of cell surface receptors, the Trk receptor tyrosine kinases and the p75 neurotrophin receptor (p75NTR). Both the transduction of survival and cell death signalling is mediated by p75NTR and pro-death signalling is presumably due to an imbalance in favor of pro-neurotrophins over proteolytically processed mature proteins [12,13]. Our and other laboratories demonstrated that genetic or pharmacological targeting of the neurotrophin interaction site or the intracellular cell death domain p75NTR resulted in beneficial effects on neurogenesis, lesion size, and neurological outcome after experimental TBI [[14], [15], [16], [17], [18], [19]].

However, various co-receptors of have been identified to modulate p75NTR-mediated signals including Trk receptors, Nogo receptor, Ephrin A, and sortilin [[20], [21], [22], [23]]. In particular, a tripartite complex of pro-neurotrophins, p75NTR and sortilin was identified that induces apoptotic signaling in neurons [23,24]. Subsequently, pro-neurotrophin induced p75NTR-sortilin mediated neuronal death has been implicated in both acute neuronal injuries and chronic neurodegenerative diseases [[25], [26], [27], [28], [29]].

Sortilin, also known as the neurotensin receptor-3, is the archetypical family member of vacuolar protein sorting 10 protein (VPS10P) domain receptors which control the subcellular fate of several proteins central to brain function [30]. Sortilin is synthesized as a proprotein, processed in the trans-Golgi network, and composed of the N-terminal VPS10P domain followed by a transmembrane domain and a short cytoplasmic tail [31,32]. Sortilin binds to a great variety of ligands and serves as a cargo receptor in different cell types and tissues. Many structural features of sortilin have been elucidated, including conformational and allosteric responses to different ligands, which partially explain how sortilin mediates various functions [[33], [34], [35]].

Several studies investigated the role of sortilin in neuronal cell death using sortilin-deficient mice. For example, reduced neuronal apoptosis was observed in the developing retina, whereas the developmental apoptosis of sympathetic or spiral ganglion neurons was not affected by sortilin-deficiency, however, sortilin prevented age-dependent degeneration of sympathetic neurons [36,37]. In the context of central nervous system (CNS) injuries, it was shown that lesioned corticospinal neurons from sortilin-deficient mice were protected from death [37]. In contrast, sortilin-deficiency was not sufficient to attenuate the loss of dorsal root ganglion (DRG) neurons after sciatic nerve injury [38] and in response to experimental autoimmune encephalomyelitis or experimental stroke, similar neurodegenerative effects were observed in sortilin-deficient mice and wild-type littermates [39]. Therefore, the role of sortilin in neuronal cell death seems to be context-dependent in terms of developmental stage, types of neurons and injury model.

The lack of effective treatments after TBI makes it necessary to identify molecular targets for novel therapeutic approaches and the p75NTR/sortilin complex represents a promising target. However, the knowledge about the role of sortilin in TBI is scarce. To address this issue, we subjected sortilin-loss-of-function mouse mutants and their wildtype littermates to the controlled cortical impact (CCI) model of TBI to investigate whether sortilin contributes to secondary brain damage. For this purpose, we examined sensorimotor deficits using a neurological severity score (NSS) and the rotarod (RR) performance test, brain damage by lesion size volumetry, cell death by TdT-mediated dUTP-biotin nick end labeling (TUNEL) staining, and calcium-mediated excitotoxicity by western blotting of spectrin breakdown products (SBDPs). Furthermore, we analysed mRNA expressions of pro-inflammatory markers, neurotrophins as well as p75NTR, VPS10 domain receptors, and the sortilin interactor progranulin.

## 2 Material and methods

### 2.1 Animals and study groups

**Fig. 1:**
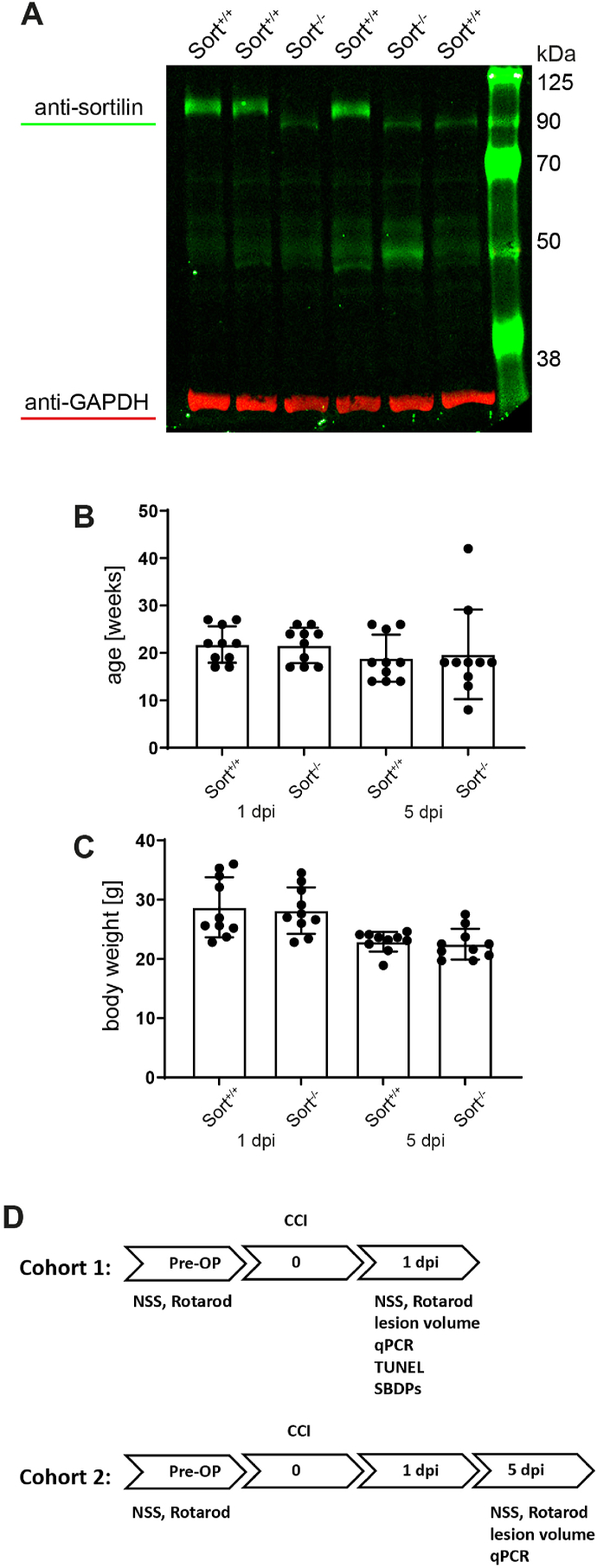
Anti-sortilin Western blot, age and body weight of examined Sort^+/+^ and Sort^−/−^mice, and study design. A protein band of about 90 kDa was detected specifically in sortilin-deficient (Sort1^−/−^) mice, consistent with the presence of a truncated protein after disruption of the reading frame in Exon 14 (A). Age (B) and body weight (C) were heterogenous without statistical significance (one way ANOVA, Šídák's multiple comparisons test). The flow chart shows the experimental time-course (D). Arrows represent days pre or post injury (dpi) after controlled cortical impact (CCI). Text above indicates interventions, text below indicates outcome parameters. Neurological severity score (NSS) and Rotarod (RR) performance were assessed one day before as well as 1 dpi (cohort 1) and 5 dpi (cohort 2). Lesion volume (LV), quantitative polymerase chain reaction (qPCR), terminal desoxynucleotidyl transferase-mediated dUTP-biotin nick end labeling (TUNEL) and spectrin breakdown products (SBDPs) were analysed at the respective time points.

All experiments were approved by the Landesuntersuchungsamt Rheinland-Pfalz (protocol number 23 177-07/G 12-1-010) and were in accordance with the German Animal Welfare Act and the ARRIVE guidelines. The B6.ICp4.Sort^1tm1Tew^ (Sort1^tm1Tew^) mouse strain with a deletion in exon14 in *Sort1* gene was used [37] (provided by A. Nykjaer, Aarhus University, Denmark). Genotypes were identified using qPCR and referred to as wild-type (Sort1^+/+^) or sortilin-deficient (Sort1^−/−^) mice [37]. Four male mice of each genotype were used for validation of Sort1 exon 14 deletion by qPCR and Western blot, where loss of the full length protein of about 110 kDa was confirmed. However, a protein band of about 90 kDa was detected specifically in Sort1^−/−^, consistent with the presence of a truncated protein after disruption of the reading frame in Exon 14 (Fig. 1A).

Until the start of the study, animals were housed in sex-matched groups of 3–5 mice per cage. During the study, mice were housed individually in filter cages to minimize the risk of wound infection and to prevent potentially detrimental dominance behavior for wound healing. The cages were equipped with embedding and nest building material, and a plastic cylinder for enrichment. Cages were enclosed in an airflow cabinet (Uniprotect, zoonlab, USA) under a 12/12 h light/dark-cycle at 22 ± 2 °C, with 55 % humidity. Animals had always ad libitum access to regular rodent diet (Cat. No. V1126-00, ssniff, Germany) and fresh water as described [40].

To address the early phase of secondary injury, we examined the time-points 1 day post injury (dpi) and 5 dpi in two separate cohort. A total of 20 Sort1^−/−^ and 20 Sort1^+/+^ mice were included. Cohort 1 included male and female animals, whereas cohort 2 included only female animals (Table 1). The sex heterogeneity was due to the limited availability of male mice, as breeding of the Sort1^tm1Tew^ mouse strain for this study resulted in an overall higher proportion of female mice than male mice regardless of genotype for unknown reasons. However, sortilin deficiency was given greater weight than sex differences.

**Table 1:** Genotype and sex characteristics of experimental cohorts.

Cohort	Groups (sample size, sex)
**1**	Sort1+/+ (n = 10, 4 male, 6 female) Sort1−/− (n = 10, 2 male, 8 female)
**2**	Sort1+/+ (n = 10, female) Sort1−/− (n = 10, female)

Mice were also heterogeneous regarding age (Fig. 1B) and body weight (Fig. 1C), however, these differences did not reach a statistically significant level. For both cohorts, a random number table with the parameters “number of groups” and “group size” was generated using a web-based random group generator by a person who was not involved in any part of the experiments or analyses. This yielded lists of pseudo-randomized numbers, that corresponded to the ear tag number of the mice, which consecutively matched each number to one of the groups.

All mice were subjected to the CCI model of TBI and examined by brain lesion volumetry, NSS, rotarod testing, and qPCR (Fig. 1D). Additionally, cohort 1 was analysed by TUNEL-staining and western blotting of SBDPs. Three naïve female mice of each genotype were added for reference purposes. All experimenters performing CCI procedure, behavioral tests and tissue preparation and analyses were blinded to the treatment groups. Data shown from p75NTR-deficient mice were obtained from tissues collected in a previous studies (animal protocol approval number 23 177-07/G12-1-010) [41].

### 2.2 CCI model of TBI

The CCI model was performed as described previously [19]. In brief, animals were anesthetized with isoflurane (induction 4 vol%, maintenance 2 vol% via mask inhalation). The head was placed in a stereotactic frame (Kopf Instruments, Tujunga, USA) and a craniotomy of 4 × 4 mm was then drilled above the right parietal cortex, and the excised bone fragment was flapped laterally with the dura mater remaining intact. The cortical impact was applied was to the right parietal cortex by a pneumatic-driven impactor (L. Kopacz, Mainz, Germany) with the following parameters: velocity 8 m/s, duration 150 ms, depth 1.0 mm. After haemostasis, the cranial bone piece was folded back and craniotomy was sealed with tissue glue (Histoacryl®, Braun, Melsungen, Germany). Skin incisions were closed with single button sutures. For maintaining physiological temperature of 37 °C, mice were kept on a feedback-controlled heating pad (Hugo Sachs, March-Hugstetten, Germany) during surgery and afterwards transferred to an incubator (Dräger, Lübeck, Germany) for 90 min. Animals regained consciousness within 10 min after the induction of trauma.

### 2.3 Neurological severity score and rotarod performance

Animals were tested one day before CCI as well as 1 and 5 dpi. NSS was used to assess motor ability, alertness, balance, anxiety, and general reflexes and the RR performance test (Panlab RotaRod, Harvard Apparatus, Holliston, MA) was used to assess sensorimotor function as previously described [42,43]. Briefly, mice were tested on an accelerating RR (height 18.5 cm) and the latency to fall was recorded. The initial RR speed was four rounds per minute (rpm), which accelerated within 5 min to a final speed of 40 rpm. Animals completed four training trials one day before CCI, followed by two trials to determine the pre-injury rotarod performance. The best value from two trials per animal and time point was analysed. Investigators were blinded for genotype identity.

### 2.4 Euthanasia and tissue preparation

After anesthesia with 4 vol% isoflurane for 1 min, animals were killed by cervical dislocation. Brains were immediately removed, cryopreserved in powdered dry ice and stored at −20 °C until further processing. 12 μm thick coronal sections at 16 consecutive levels were cut for cresyl violet and TUNEL-Staining using a cryotome (HM 560 Cryo-Star, Thermo Fisher Scientific) and collected in 500 μm intervals on glass slides (Superfrost Plus, Menzel GmbH, Brauschweig, Germany) from Bregma +3.14 mm to −4.36 mm according to Paxinos and Franklin's mouse brain atlas and stored at −20 °C. During the cryosectioning process sections containing the lesioned tissue were collected for RNA or protein extraction from Bregma +0.64 mm to −2.86 mm as described [43].

### 2.5 Brain lesion volumetry

Brain lesion volumetry was performed after cresyl violet staining with 16 consecutive sections per mouse brain and images were taken using a stereo microscope (Stemi 305, Zeiss, Oberkochen, Germany) by investiagtors blinded for genotype identity. The brain lesion was defined as unstained area and was determined along with the ipsi- and contralesional hemisphere volume using ZEN imaging software tools (RRID:SCR_013672, Zeiss, Oberkochen, Germany) by investigators blinded for genotype identity. Brain lesion volume was calculated by summation of areas multiplied by the distance between sections using the following formula: $V=\sum_{1}^{16}Ax*500\;\mu m$ and expressed relative to the ipsilesional hemisphere volume [19].

### 2.6 TUNEL staining

TUNEL staining was performed using 12 μm thick cryosections, post-fixated for 5 min with 4 % paraformaldehyde, using an in situ cell death detection kit (Roche Molecular Biochemicals, Indianapolis, IN) in combination with 4′,6-diamidino-2-phenylindole (DAPI) solution (0.5 μg/ml in phosphate buffered saline) according to the manufacturers instructions. Images were taken with an Axiovert microscope using a 20x objective (Zeiss, Germany). The number of TUNEL+ and DAPI + nuclei were counted using ImageJ, and expressed as the TUNEL+/DAPI + ratio.

### 2.7 Quantitative real-time PCR (qRT-PCR)

RNA extraction and cDNA synthesis were performed using RNeasy Kit and QuantiTect Reverse Transcription Kits (both Qiagen, Venlo, Netherlands) from cryosections containing lesioned and perilesional regions [43]. Equal amounts of cDNA were amplified by qRT-PCR using DyNAmoTM ColorFlash Probe qPCR for *Ngf* and *Bdnf*, Maxima Probe qPCR Master Mix for *Sort1* (both Thermo Fisher Scientific, Scherte, Germany), LightCycler 480 Probes Master for *Musort1* (Hoffmann-La Roche, Basel, Switzerland), QuantiNova-Qiagen for *Ngfr*, and *Il6* (Qiagen N.V., Venlo, Netherlands), and Absolute Blue qPCR SYBR Green Mix (Thermo Fisher Scientific, Waltham, MA) for the remaining targets. Samples were analysed in duplicates using the LightCycler Software, Version 4.5 (F. Hoffmann-La Roche AG, Basel, Switzerland). Quantification was performed using a target specific standard curve and normalization to the reference gene cyclophilin A (Ppia) as described [44]. Primer sequences, amplification products lengths, and gene accession numbers are summarized in Table 2.

**Table 2:** Primer sequences and amplicon sizes.

Target Gene	Amplicon size (bp)	Oligonucleotide sequence (5’ – 3’)	Gene Bank No.
*Ppia*	146	F: GCGTCTSCTTCGAGCTGTT	NM_008907
		R: RAAGTCACCACCCTGGCA	
*mIl6*	471	F: TCGTGGAAATGAGAAAAGAGTTG	NM_031168
		R: TATGCTTAGGCATAACGCACTAG	
		CY5: TGCTCTCCTAACAGATAAGCTGGAGTCAC-PH	
		FL: CATAAAATAGTCCTTCCTACCCCAATTTCC-FL	
*Tnfa*	212	F: TCTCATCAGTTCTATGGCCC	NM_013693
		R: GGGAGTAGACAAGGTACAAC	
*Iba 1*	144	F: ATCAACAAGCAATTCCTCGATGA	NM_019467
		R: CAGCATTCGCTTCAAGGACATA	
*Gfap*	120	F: CGGAGACGCATCACCTCTG	NM_001131020
		R: TGGAGGAGTCATTCGAGACAA	
*Bdnf*	155	F: ACTTGGCCTACCCAGGTG	NM_007540
		R: GTTGGGCCGAACCTTCT	
		CY5: GACACTTTTGAGCACGTCATCGAAGAGCTG-PH	
		FL: AGAGGTCTGACGACGACATCACTGGC-FL	
*Ngf*	218	F: CGGGCAGCTTTTTGGAA	NM_013609
		R: ACCTCACTGCGGCCAGTA	
		CY5: CCCAATAAAGGTTTTGCCAAGGACGC-PH	
		FL: ACCCAAGCTCACCTCAGTGTCTGG-FL	
*Ntf3*	115	F: AGTTTGCCGGAAGACTCTCTC	NM_001164034
		R: GGGTGCTCTGGTAATTTTCCTTA	
*mGrn*	145	F: ATGCTGTGTGCTGTGAGGAC	NM_008175
		R: CACTCCACATTCCCAACCTT	
*Ngfr*	288	F: GGGGTGGGCTCAGGACT	NM_033217
		R: TATGAGGTCTCGCTCTGGAGGT	
		CY5: CCGAATGCGAGGAGATCCCTGGCC-PH	
		FL: GTGCACGCCCTGGGCTGAC-FL	
*Sorcs2*	174	F: GTCTCGCTCATCAGCACGTC	NM_030889
		R: TGTCCCAAAATCTGAGGACCT	
*Sorl1*	163	F: AGTCGAGACTCCCTTTCCTATTC	NM_011436
		R: TGCGTTCCTAGCCGGAGAT	
*Sort1*	376	F: GCTCTCAGAAGCATTCACAC	NM_001271599
		R: TTCCATTCAATTCACGGCAC	
		CY5: TGGCTGGTGGGCATAGTTATTCTC-PH	
		FL: GCAGCCTTGGAGCATCATAAGC-FL	
*Musort1*	236	F: ATCCTGGACTCTGGAGGCAT	NM_001271599
		R: CGTGGTATAGTCATCCTCTTCACAAT	
		CY5: GAGCCAGGTCCATGAACATCAGCATC-PH	
		FL: GATTCACAGAGTCTTTCATTACCCGCCA-FL	

### 2.8 Western blotting

Proteins were extracted from the upper quadrant of ipsilesional or corresponding non-injured coronal brain slices (in total 40 sections of 30 μm for each brain) obtained during serial cryosectioning from Bregma +0.64 mm to −2.86 mm according to a mouse brain atlas (Mouse Brain in Stereotaxic Coordinates, 3rd Edition, Franklin & Paxinos) followed by immunoblotting as described [43]. The following primary antibodies were used: mouse anti-αII-spectrin (1:1000, BML-FG6090, Enzo Life Science, Farmingdale, USA), mouse anti-GAPDH (1:2000, ACR001PS, Acris Antibodies Inc., San Diego, USA), rabbit anti-sortilin (1:500, ANT-009, Alomone Labs, Jerusalem, USA), followed by incubation with appropriate species-specific secondary infrared dye-conjugated antibodies: goat anti-rabbit IgG (1:15000, IRDye 800CW, Li-Cor Bioscience, Bad Homburg, Germany), goat anti-mouse IgG (1:15000, IRDye 680RD, Li-Cor Bioscience, Bad Homburg, Germany). Protein band densities were analysed using the Odyssey SA Imaging System and quantified with Image Studio (RRID:SCR_014579; LI-COR). Data was normalized to GAPDH expression of each sample.

### 2.9 Statistical analysis

All data were analysed using GraphPad Prism software (version 9, GraphPad Software Inc., San Diego, California, USA). Outliers were identified by the ROUT's method (Q = 1 %) and excluded from further analysis. Distribution was analysed by Shapiro-Wilk normality test, Kolmokorov-Smirnov test and QQ plots. Comparative analysis was performed depending on data distribution by ordinary one-way analysis of variance (ANOVA) followed by Šídák's adjustment for multiple comparisons or students t-test for normally distributed data, Kruskal-Wallis test followed by followed by Dunn's adjustment for multiple comparisons, or Mann-Whitney *U* test when data failed normality tests. Behavioral testing was analysed using two-way ANOVA followed by Šídák's adjustment for multiple comparisons. Values are presented as mean ± standard deviation (SD); p < 0.05 was considered as significant.

## 3 Results

### 3.1 Sortilin protein and mRNA expression is reduced after CCI

**Fig. 2:**
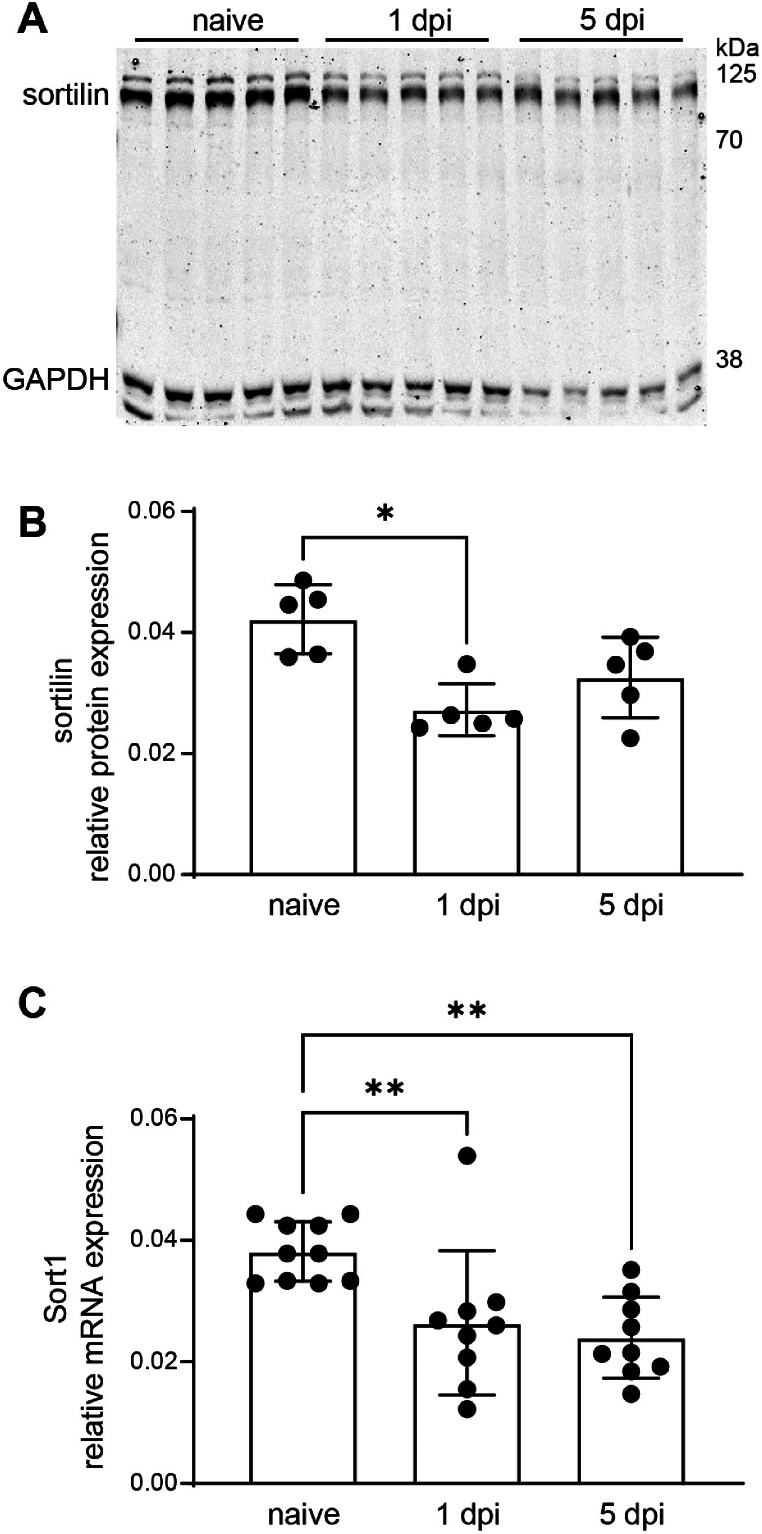
Sortilin protein and mRNA expression is reduced after CCI. Timeline of posttraumatic sortilin regulation in perilesional cortical brain tissue was measured by Western blot analysis (A, B) and real-time qPCR (C). Protein expression was normalized to the reference protein glycerinaldehyd-3-phosphat-dehydrogenase (GAPDH) and was reduced 1 day post injury (dpi; A,B). mRNA expression was normalized to the reference gene cyclophilin A (Ppia) and was reduced 1 and 5 dpi (C). Data are expressed as mean ± SD, individual data is shown as dots. Kruskall-Wallis test, Dunn's multiple comparison test,*p < 0.05; **p < 0.01.

Initially, we examined the brain mRNA and protein expression of sortilin in C57BL/6 mice at 1 dpi and 5 dpi, time-points characterized by neuronal loss and massive inflammatory reactions, in our CCI model of TBI [45,46]. CCI caused the reduction of relative sortilin protein levels at 1 dpi, but no statistically significant effects was observed at 5 dpi (Fig. 2A and B). The relative sortilin mRNA expression was significantly reduced, both at 1 dpi and 5 dpi (Fig. 2C).

### 3.2 CCI induces sensorimotor deficits irrespectively of sortilin-deficiency

**Fig. 3:**
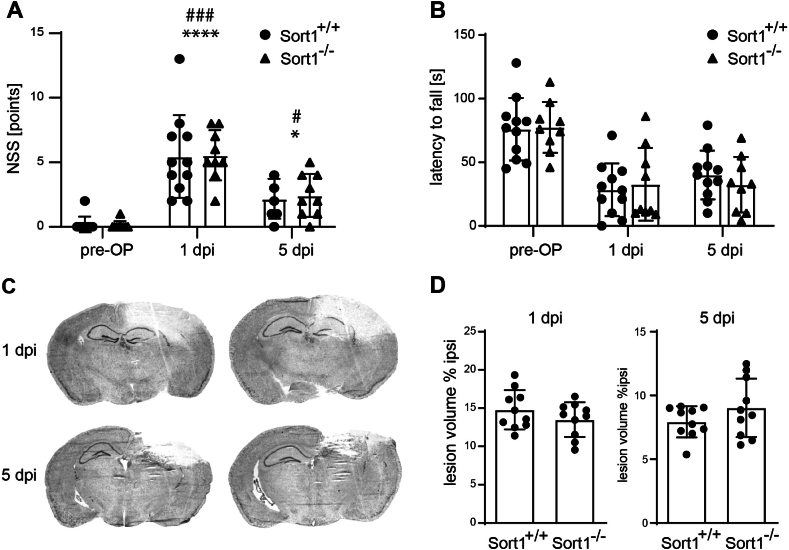
Sortilin-deficiency does not affect CCI-induced sensorimotor deficits and brain lesion volume. Sensorimotor function was investigated by a neurological severity score (NSS; A). * indicates significant differences between 1 dpi or 5 dpi and pre-OP, # indicates significant differences between 1 dpi and 5 dpi. Sensorimotor function (expressed as latency to fall) was tested in a RotaRod analysis (B). NSS and Rotarod revealed no difference between Sort1^+/+^ and Sort1^−/−^ mice. Data are expressed as mean ± SD, individual data is shown as dots, Two-way ANOVA, Šídák's multiple comparisons test. Representative cresyl-violet stained sections at 1 and 5 days post injury (dpi) at the coronal plane (C). Lesion volume of sortilin deficient (Sort1^−/−^) mice was not different from wildtype (Sort1^+/+^) mice 1 dpi and 5 dpi (D). Data are expressed as mean ± SD, individual data is shown as dots, Student's *t*-test.

To evaluate the relevance of sortilin in the early phase of TBI, we subjected two cohorts of Sort1^*+/+*^ and Sort1^*−/−*^ mice to CCI and set the survival time to 1 dpi or 5 dpi (Fig. 1). Sensorimotor deficits were assessed using NSS and the RR performance test [47]. Mice showed CCI-induced sensorimotor deficits, as demonstrated by increased NSS and decreased RR performance at both post-traumatic time points (Fig. 3A and B). However, no differences in sensorimotor deficits were observed between Sort1^*+/+*^ and Sort1^*−/−*^ mice at 1 dpi or 5 dpi.

### 3.3 Sortilin-deficiency does not affect brain lesion size after CCI

Brain sections were stained with cresyl violet to visualize the extent of brain tissue damage which approximately doubles within 24 h after the primary injury in our CCI model of TBI [46]. Brains from Sort1^*+/+*^ and Sort1^*−/−*^ mice exhibited marked lesions comprising cortical and subcortical brain regions (Fig. 3C), both at 1 dpi and 5 dpi. To quantify the extent of brain lesions, they were delineated over consecutive sections, and the relative lesion volume was calculated. This brain volumetric analysis revealed similar lesion volumes between Sort1^*+/+*^ and Sort1^*−/−*^ mice (Fig. 3D).

### 3.4 CCI-induced cell death and production of SBDPs are not attenuated by sortilin-deficiency

**Fig. 4:**
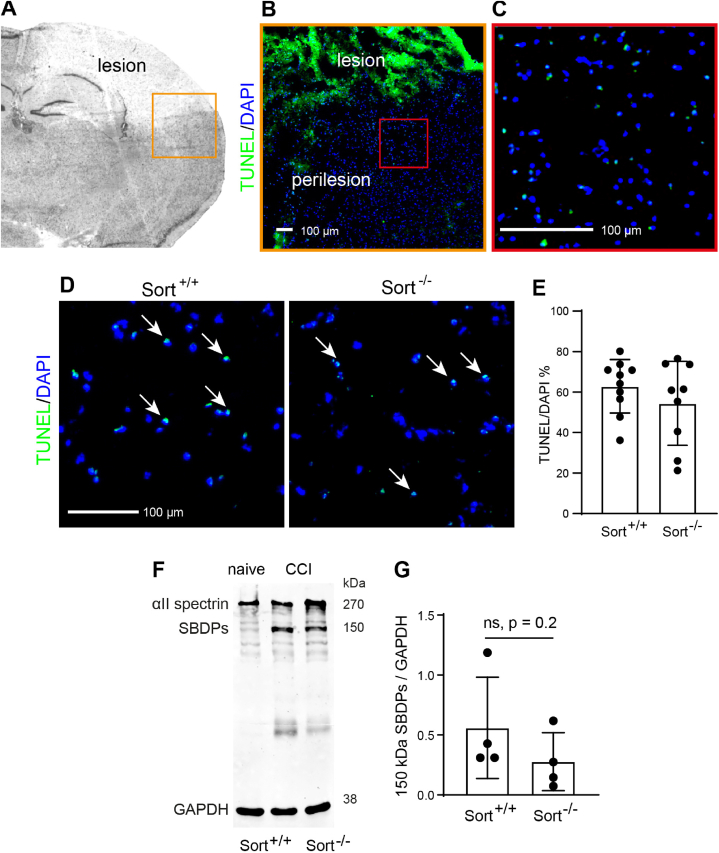
Sortilin-deficiency does not affect CCI-induced cell death and production of SBDPs. (A–C) Images illustrating the location of cell death examination by TUNEL staining at 1 dpi (A) Ipsilesional hemisphere of a cresyl violet stained brain cryosection. The brain lesion does not show cresyl violet staining and the orange box includes lesional and perilesional regions. (B) Low-magnification fluoresence image showing TUNEL (green) and DAPI (blue) staining corresponding to the location of the orange box. The lesion area can be identified by a high-intensity TUNEL staining and extensive loss of DAPI staining due to tissue destruction. The perilesional area contains intact cell nuclei identified by DAPI, which partially co-localize with TUNEL stainining. (C) High-magnification fluorescence image corresponding to the location of the red box shows the region of interest in the deep layers of the perilesional cortex for TUNEL/DAPI staining analysis. (D) Representative images of TUNEL/DAPI staining in brain cryosections from Sort^+/+^ mice and Sort^−/−^ mice. Some TUNEL/DAPI co-stained cells are marked by arrows. (E) Quantification of the TUNEL/DAPI ratio. Data are expressed as mean ± SD, individual data is shown as dots, Student's *t*-test. (F) Western blot showing spectrin breakdown products (SBDPs) at ∼150 kDa at 1 dpi in protein lysates of the ipsilesional brain tissue from Sort^+/+^ mice (lane 2) and Sort^−/−^ mice (lane 3) as well as in naïve Sort^+/+^ mice (lane 1; C). (G) Quantification of SBDPs in ipsilesional brain tissue. Data are expressed as mean ± SD, individual data is shown as dots, Mann Whitney test.

To examine the CCI-induced brain tissue damage at the cellular level, we performed TUNEL staining along with DAPI counterstaining at 1 dpi. In agreement with previous work [46], we observed substantial numbers of TUNEL^+^/DAPI^+^ cells at this time-point in the brain lesion core both in Sort1^*+/+*^ and Sort1^*−/−*^ mice (Fig. 4A). Calculating the ratio of TUNEL+/DAPI + cells indicated a similar extent of cell death in Sort1^*+/+*^ and Sort1^*−/−*^ mice (Fig. 4B). Using western blots probed with antibodies specific to αII-spectrin (Fig. 4C), we also compared the production of SBDPs between Sort1^*+/+*^ and Sort1^*−/−*^ mice as a proxy for calcium-mediated excitotoxicity [48]. Full length αII-spectrin was cleaved to SBDPs after CCI with a major protein band migrating at about 150 kDa after SDS-PAGE (Fig. 4C). The protein band densities of the SBDPs were statistically not different between Sort1^*+/+*^ and Sort1^*−/−*^ mice (Fig. 4D, p = 0.2, Mann-Whitney *U* test).

### 3.5 Sortilin deficiency does not alter genotype-specific mRNA expression

**Fig. 5:**
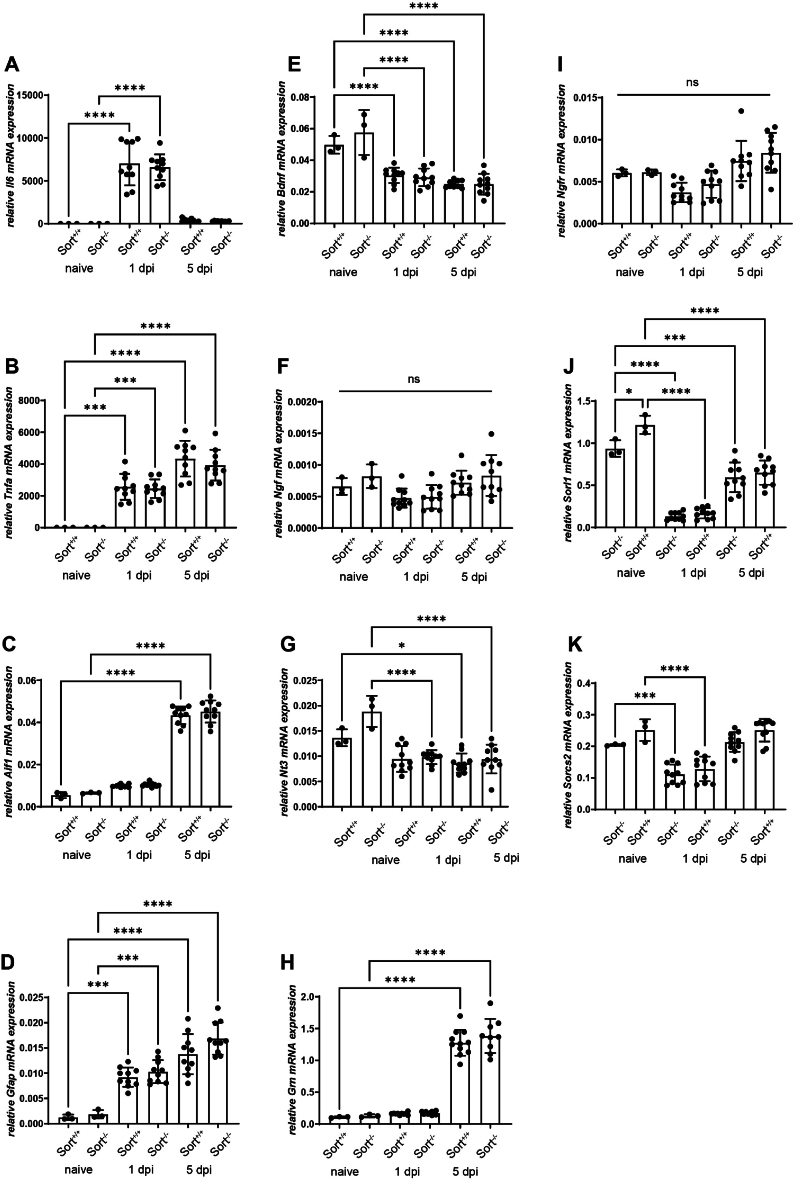
Sortilin deficiency does not alter genotype-specific mRNA expression. Quantitative assessment of the mRNA expression of inflammatory marker genes (A–D), neurotrophic factors (E–G), the sortilin interactor progranulin (H) and receptors (I–K), in naïve mice as well as 1 and 5 days post injury (dpi). Data was normalized to the reference gene cyclophilin A (Ppia). Inflammatory markers were increased after CCI, but were not different between sortilin deficient (Sort^−/−^) and wildtype (Sort^+/+^) mice (A–D). Neurotrophic factors were reduced after trauma with the exception of no regulation of Ngf (F), but revealed no difference between genotypes (E–G). Progranulin was increased 5 dpi, but irrespectively of the genotype (H). The p75 neurotrophin receptor was not regulated by trauma or genotype (I), whereas the sortilin related receptor 1 (SORL 1, J) and sortilin related VPS10 Domain Containing Receptor 2 (SORCS2, K) were reduced after trauma but affected by genotype. Data are expressed as mean ± SD, individual data is shown as dots, One way ANOVA, Šídák's multiple comparisons test, *p < 0.05; **p < 0.01; ***p < 0.001; ****p < 0.0001.

TBI triggers a rapid and pronounced inflammatory response from brain-resident microglia and astrocytes as well as infiltrating immune cells [49]. To examine the inflammatory response in Sort1^*+/+*^ and Sort1^*−/−*^ mice, we determined gene expression levels of the acute phase markers IL-6 and TNF-α, the microglia marker Iba-1 (encoded by the *Aif1* gene), and the reactive astrocyte markler GFAP in ipsilesional tissue samples by qPCR. All these gene markers were significantly up-regulated after CCI at 1 dpi and/or 5 dpi. However, none of them was differentially expressed between Sort1^*+/+*^ and Sort1^*−/−*^ mice (Fig. 5A–D).

Next, gene expression levels of the sortillin-associated neurotrophic factors and receptors BDNF, NGF, NT3, progranulin (encoded by the *Grn* gene), and p75NTR (encoded by the *Ngfr* gene) as well as the VPS10-related sortilin family members SORL1 and SORCS2 were determined. We observed some CCI-induced gene expression regulations, however, irrespectively of the Sort1^*+/+*^ and Sort1^*−/−*^ genotypes (Fig. 5E–KH). Taken together, several CCI-regulated inflammation- and sortilin-associated gene markers were not differentially expressed between Sort1^*+/+*^ and Sort1^*−/−*^ mice at 1 dpi and/or 5 dpi.

## 4 Discussion

In the present study, we tested the hypothesis that the p75NTR co-receptor sortilin is critically involved during the early phase after TBI. To this end, we subjected two cohorts of sortilin-deficient mice and wild-type littermate mice to the widely used CCI model of TBI and investigated them at 1 dpi and 5 dpi. We examined sensorimotor deficits, the histopathological brain damage, cell death markers, and the expression of key inflammatory markers. Altogether, our results suggest that sortilin is dispensable for secondary injury processes in the murine CCI model of TBI.

The data raise questions about the contextual role of sortilin in TBI and other types of CNS injuries. While there is consensus about the role of the p75NTR-Sortilin axis in the developing nervous system [11,37,[50], [51], [52], [53]], data are less consistent in the context of acute brain injuries. For example, after subarachnoid hemorrhage (SAH) in rats, evidence was provided that sortilin mediates both beneficial [54] or deleterious effects [55]. The beneficial effects have been attributed to sortilin interaction with recombinant progranulin in a therapeutic approach [54], which are probably mediated via endocytic internalization and delivery to the lysosomal compartment [56]. Interestingly, a recent study indicated that sortilin-mediated internalization of progranulin occurs predominantly in neurons rather than in microglia [57]. However, we and others have found both neuroprotective and anti-inflammatory effects of progranulin after TBI [[58], [59], [60]]. These findings suggest that sortilin-mediated internalization of progranulin or other soluble factors may not only affect neurons but also modulate glial and immune function, which would be in line with the expression of sortilin in various glia and immune cell types (www.proteinatlas.org).

Studies provided evidence that reduction of sortilin levels has a positive effect on neuronal survival in animal models of brain injury [55,61] and in vitro [62]. Furthermore, increased levels of sortilin have been associated with neuronal cell death [63,64]. Assuming that sortilin exerts predominantly cell death promoting effects, the CCI-induced downregulation of sortilin may be an adaptive process in response to injury. However, there are also studies consistent with our results showing that sortilin is dispensable for p75NTR-mediated cell death. For example, sortilin seemed dispensable for the induction of cell death of DRG neurons [38], which suggests that p75NTR-dependent loss of DRG neurons involves sortilin-independent pathways.

We also asked whether a compensatory regulation of p75NTR may explain the lack of neuroprotection in sortilin-deficiciency in our experimental setting. However, we could not detect a compensatory mRNA expression of p75NTR in sortilin-deficient mice. In contrast, our previous data showed that nerve growth factor receptor (NGFR)-deficient mice, lacking the proneurotrophin-binding site of p75NTR, had an increased sortilin mRNA expression after CCI [19]. The TBI-induced reduction of sortilin expression in Sort1^+/+^ mice, as shown in the time-course, could potentially diminish sortilin-specfic differences between Sort1^+/+^ and Sort1^−/−^ mice.

Sortilin and related proteins have been reported to bind several ligands [23,37]. Due to the construction of the mouse mutant used in this study with reading frame disruption in Exon 14, there is a possibility that a soluble truncated sortilin protein is still expressed in this mouse line. Indeed, we detected a protein band of about 90 kDa in sortilin-deficient mice which was absent in WT. However, we were unable to verify protein identity. It is tempting to speculate that a truncated sortilin protein may have contributed to the lack of any genotype-dependent differences in our study. A previously identified surface exposed epitope can bind to pro-neurotrophins in vitro [65] is upstream of the exon 14 deletion and pro-neurotrophins have been described as ligands of p75NTR-induced neuronal cell death [18,[66], [67], [68]].

Sortilin also functions as the lysosomal trafficking receptor for progranulin [69]. Mice lacking sortilin had elevated levels of progranulin [56]. While there was no difference in progranulin mRNA expression levels between sortilin deficient mice and their WT littermates in our model of TBI, it may be possible that high levels of progranulin protein at 5 dpi competed with sortilin recuitment to the p75NTR complex by binding to sortilin for lysosome formation and that, therefore, less sortilin was available for formation of the p75NTR-sortilin-receptor-complex for pro-neurotrophin binding.

This study has limitations that need to be taken into account. Mice were heterogenous regarding age, although this did not reach statistical significance, and gender. Both age and gender differences are known to have an impact on neuronal death after acute brain injuries [67,70]. However, females outnumbered the males for the timepoint 1 dpi and for the timepoint 5 dpi only female mice were available. Regarding the age differences, a previous publication of our laboratory using the same model of CCI demonstrated that differences in histopathological injury and contusion volume were not different between young and aged mice after 24 and 72 h [44]. This suggests that histopathological results were most likely not significantly affected by differences in age.

Behavioural assessments were limited to an observational test (neurological severity score) and a sensorimotor test (Rotarod performance). The examination of CCI-induced cognitive deficits was not included in this study due to the short post-traumatic recovery times of 1 dpi and 5 dpi, making it difficult to investigate cognitive effects without possible interference by sensorimotor deficits and testing.

As a further limitation, no transcriptomics or proteomics technologies were used and thus we do not have an overview of all the mRNA or protein expression changes. In addition, immunohistochemistry may have revealed genotype-dependent differences in the distribution of proteins or mRNAs examined in this study by Western Blot or qPCR, respectively.

Therefore, not all potential changes induced in the p75NTR sortilin axis were targeted by our investigation. However, because the results clearly show no effect of sortilin-deficiency in terms of sensorimotor deficits, brain histopathology, and inflammatory as well as cell death markers, the significance of potential mRNA or protein expression changes remains questionable.

In conclusion, our results suggest that sortilin is a modulatory rather than an essential factor in the acute and early brain tissue response after TBI.

## Data availability statement

Data will be made available on request.

## Ethics approval and consent to participate

No human subjects. All animal experiments were approved by the Landesuntersuchungsamt Rheinland-Pfalz (protocol number 23 177-07/G 12-1-010) and comply with the ARRIVE guidelines.

## Consent for publication

Not applicable.

## Funding

Institutional funding

## CRediT authorship contribution statement

**Irina Staib-Lasarzik:** Writing – original draft, Visualization, Validation, Data curation. **Christina Gölz:** Visualization, Validation, Methodology, Investigation. **Wieslawa Bobkiewiecz:** Methodology, Investigation. **Pawit Somnuke:** Visualization, Validation, Investigation. **Sebastiani Anne:** Methodology, Investigation. **Serge C. Thal:** Supervision, Project administration, Methodology, Conceptualization. **Michael K.E. Schäfer:** Writing – review & editing, Writing – original draft, Supervision, Project administration, Methodology.

## Declaration of competing interest

The authors declare no competing interests or conflict of interest.
